# Factors Associated With Response to Pilot Home-Based Light Therapy for Fatigue Following Traumatic Brain Injury and Stroke

**DOI:** 10.3389/fneur.2021.651392

**Published:** 2021-07-15

**Authors:** Laura J. Connolly, Shantha M. W. Rajaratnam, Gershon Spitz, Steven W. Lockley, Jennie L. Ponsford

**Affiliations:** ^1^Monash Epworth Rehabilitation Research Centre, Epworth Healthcare, Melbourne, VIC, Australia; ^2^School of Psychological Sciences, Turner Institute for Brain and Mental Health, Monash University, Melbourne, VIC, Australia; ^3^Division of Sleep and Circadian Disorders, Departments of Medicine and Neurology, Brigham and Women's Hospital, Boston, MA, United States; ^4^Division of Sleep Medicine, Harvard Medical School, Boston, MA, United States

**Keywords:** traumatic brain injury, stroke, light therapy, fatigue, sleep disturbance, sleepiness

## Abstract

**Background:** Fatigue and sleep disturbance are common and debilitating problems after brain injury. Light therapy shows promise as a potential treatment. We conducted a trial of in-home light therapy to alleviate fatigue and sleep disturbance. The aim of the current study was to identify factors moderating treatment response.

**Methods:** Participants were 24 individuals with traumatic brain injury (TBI) (*n* = 19) or stroke (*n* = 5) reporting clinically significant fatigue. Outcomes included fatigue on Brief Fatigue Inventory (primary outcome), sleep disturbance on Pittsburgh Sleep Quality Index, reaction time (RT) on Psychomotor Vigilance Task and time spent in productive activity. Interactions of demographic and clinical variables with these outcomes were examined in linear mixed-model analyses.

**Results:** Whilst there were no variables found to be significantly associated with change in our primary outcome of fatigue, some variables revealed medium or large effect sizes, including chronotype, eye color, injury severity as measured by PTA, and baseline depressive symptoms. Chronotype significantly moderated sleep quality, with evening chronotype being associated with greater improvement during treatment. Injury type significantly predicted mean RT, with stroke participants exhibiting greater post-treatment reduction than TBI. Age significantly predicted productive activity during Treatment, with younger participants showing stronger Treatment effect.

**Conclusion:** Light therapy may have a greater impact on sleep in younger individuals and those with an evening chronotype. Older individuals may need higher treatment dose to achieve benefit.

**Clinical Trial Registration:** www.anzctr.org.au, identifier: ACTRN12617000866303.

## Introduction

Fatigue and sleep disturbance are debilitating sequelae of traumatic brain injury (TBI) and stroke across the spectrum of severity. These symptoms are reported in 30–70% of cases and impact significantly on daily activities and quality of life (1–4). Frequent sleep disturbances include excessive daytime sleepiness, hypersomnia, insomnia, reduced sleep efficiency, changes to sleep timing and sleep apnea (5, 6). Causes are thought to be multi-factorial, including both injury-related factors and secondary factors such as depression and pain (7, 8).

Pharmacological interventions have not provided long-term solutions to these problems (9–11). Recent studies have, however, offered evidence that light therapy may be an effective avenue for treatment (12–16). In a recent pilot randomized controlled trial involving 24 individuals with TBI or stroke and significant fatigue, we showed that in-home light therapy, consisting of daytime blue-enriched white light (CCT >5,000 K) and blue-depleted light (≤ 3,000 K) 3 h prior to sleep, showed positive trends in impacting fatigue, significantly reduced sleep disturbance and insomnia symptoms, and improved psychomotor vigilance and productive daily activity, relative to a control lighting condition (17). Considerable inter-individual variability was observed in responses to the intervention. There is therefore a need to identify which factors moderated response to the intervention, so that treatment may be targeted toward individuals most likely to benefit.

Only a few studies have examined factors associated with response to light therapy, for the treatment of delayed sleep-wake phase disorder (DSWPD) (18) and seasonal affective disorder (SAD) (19–21). These studies found that mood and sleep-related factors predicted the efficacy of light therapy, with one study finding that clinical effectiveness of bright light treatment (2 h of 2,500–10,000 lux light daily for 7–14 days) (based on the Hamilton Depression Rating Scale) was associated with a greater relative dominance of atypical symptoms of depression prior to treatment, which included symptoms such as hypersomnia, afternoon or evening slump, worse mood in evenings, and carbohydrate craving (19). Age has also been identified as a predictor, with younger age associated with greater treatment response to morning bright light therapy (2,500 lux, 2 h in mornings for 2 weeks) for SAD (21). Conversely, diagnosis of a personality disorder (20) and moderate consumption of alcohol (22) render an individual with SAD less likely to respond positively. Sex differences in response to light therapy have also been observed, with men demonstrating a stronger response to blue-enriched white light (6,500 K, 40 lux, 2 h) in the late evening, indicated by superior sustained attention performance and increased frontal NREM sleep slow-wave activity (23). There has been limited examination of factors associated with light therapy efficacy after brain injury (13–16, 24). In a small pilot study, we previously showed that reductions in fatigue and daytime sleepiness due to morning blue lightbox therapy were not associated with demographic, injury or cognitive characteristics of participants with TBI (12). This present study sought to extend these previous findings by investigating factors that moderate response to in-home light therapy in a larger cohort of TBI and stroke patients.

A number of potential predictive factors were selected a priori on the basis of their known influence on fatigue, sleep and sensitivity to light. Firstly, injury-related factors, such as time post-injury, injury severity, and injury type may moderate treatment response. Recovery is typically greatest in early stages (25), and functional recovery greater in individuals with less severe injury (26), so it is plausible that those with less time post-injury and less severe injury (measured by shorter post-traumatic amnesia duration; PTA), may show greater response to light therapy. Although the causes of fatigue and sleep disturbance after both TBI and stroke are not well-established, and likely multi-factorial, it is conceivable that there may be differences in response to intervention according to injury cause. Biological factors may also be associated with treatment response, including eye color, use of antidepressant medication and chronotype. There is evidence that lighter iris color (27) and antidepressant medication increase the sensitivity of the circadian system to light (28). Greater evening chronotype individuals have also been found to be more responsive to light therapy in groups with depression and bipolar disorder (29, 30). Lastly, an individual's work status (unemployed, working part time or full-time) is likely to correlate to the amount of time spent in the home, and thus the magnitude of exposure to the light therapy, which may in turn moderate treatment response (31).

The aim of the current study was to investigate which factors moderated response to in-home light therapy for individuals with fatigue following TBI and stroke. Predictive factors included level of baseline fatigue, daytime sleepiness and depressive symptoms, age, sex, injury severity measured by post-traumatic amnesia (PTA), time post-injury, injury type (TBI vs. stroke), chronotype, eye color, work status (not working, part time or full time), and use of antidepressant medication. Outcomes included measures of fatigue, sleep disturbance, psychomotor vigilance and productive activity, which were the main outcomes measured in the intervention. A priori, it was hypothesized that younger age, male sex, shorter PTA, less time since injury, injury type (TBI vs. stroke), greater evening chronotype score, unemployed work status, and current use of antidepressant medication would be associated with greater treatment response.

## Method

### Trial Design

This study was a secondary analysis of a randomized within-subject, crossover controlled trial evaluating the efficacy of light therapy for fatigue in patients with TBI or stroke, termed acquired brain injury (ABI). A power analysis [G^*^Power (32)] undertaken with power (1-β) set at 0.80 [with α = 0.05; (33)] to detect a medium effect size (*dz* = 0.60) showed a required sample size (within-subjects) of 24. The study was approved by the human research ethics committees at Epworth Healthcare (#EH2016-164) and Monash University (#9246). The study was conducted from August 2017 until June 2020.

### Light Therapy Treatment

The in-home lighting intervention consisted of blue-enriched high-intensity white light with a correlated color temperature (CCT) of approximately >5,000 K for use during the day. In the evening, for 3 h prior to sleep, the light intensity was reduced, and blue-depleted white light was used (≤ 3,000 K). Current lighting was assessed prior to study commencement. Priority was given to rooms in which a participant spent the most time (typically living space, kitchen, bedroom and ensuite bathroom). Participants were asked to keep the light schedule as stable as possible day-to-day, and the timing of each element of light exposure was based on individual sleep patterns. The specific lighting fixtures and lamps used were integrated with participants' existing lighting arrangements and a qualified electrician changed the lighting. Participants were educated on how to use and time the lights for each condition. There was no prescribed time for the light therapy, but participants were instructed to use the appropriate Treatment lighting when in their home. Light-emitting devices (i.e., phone, iPad, computer) also had system settings modified to reduce exposure to short-wavelength light during evenings. In the sham control condition, researchers changed the lamps as per the Treatment condition but the lamps did not change in color temperature from the participants' normal lighting (typically 3,000–4,000 K).

A Colormunki Light Meter (X-Rite, Grand Rapids, MI, USA) was used to measure participants' home lighting conditions and data analyzed using f.luxometer software (f.lux, Los Angeles, CA, USA). The pilot RCT paper (17) showed that during daytime Treatment conditions, the melanopic illuminance significantly increased by ~55%, from 226 ± 143 melEDI [melanopic Equivalent Daylight (D65) Illuminance] lux during the Control to 350 ± 225 melEDI lux. Meanwhile, photopic lux was maintained [Treatment (441 ± 267 lux) and Control (394 ± 216 lux)]. During nighttime Treatment conditions, there was a ~20% overall reduction in melanopic illuminance to 96 ± 65 melEDI lux, from 139 ± 77 melEDI lux during Control, and a significant reduction in photopic illuminance (Treatment: 210 ± 152 lux vs. Control: 272 ± 132 lux). Participants reported average compliance rates of 81% during the Treatment phase, in terms of utilizing the correct lights, and transitioning from day to nighttime at the designated hour. This methodology is further described in a case study paper (Connolly et al., submitted).

### RCT Protocol

The protocol for each participant was 5.5 months in duration, including a baseline of 2 weeks, and two conditions each of 2 months duration (Treatment and Control, counterbalanced), followed by a 1-month follow-up period. The study employed a cross-over design and thus all participants were exposed to both lighting conditions in randomized order, and served as their own controls. Primary and secondary outcome measures were administered at baseline and at monthly intervals (mid- and end of Treatment/Control condition), and at one-month follow-up, resulting in 6 assessment points. Assessments were conducted by a researcher who was blinded to the nature of light intervention being received. The RCT methodology and results are further detailed in another paper (17).

### Participants

**Figure 1:**
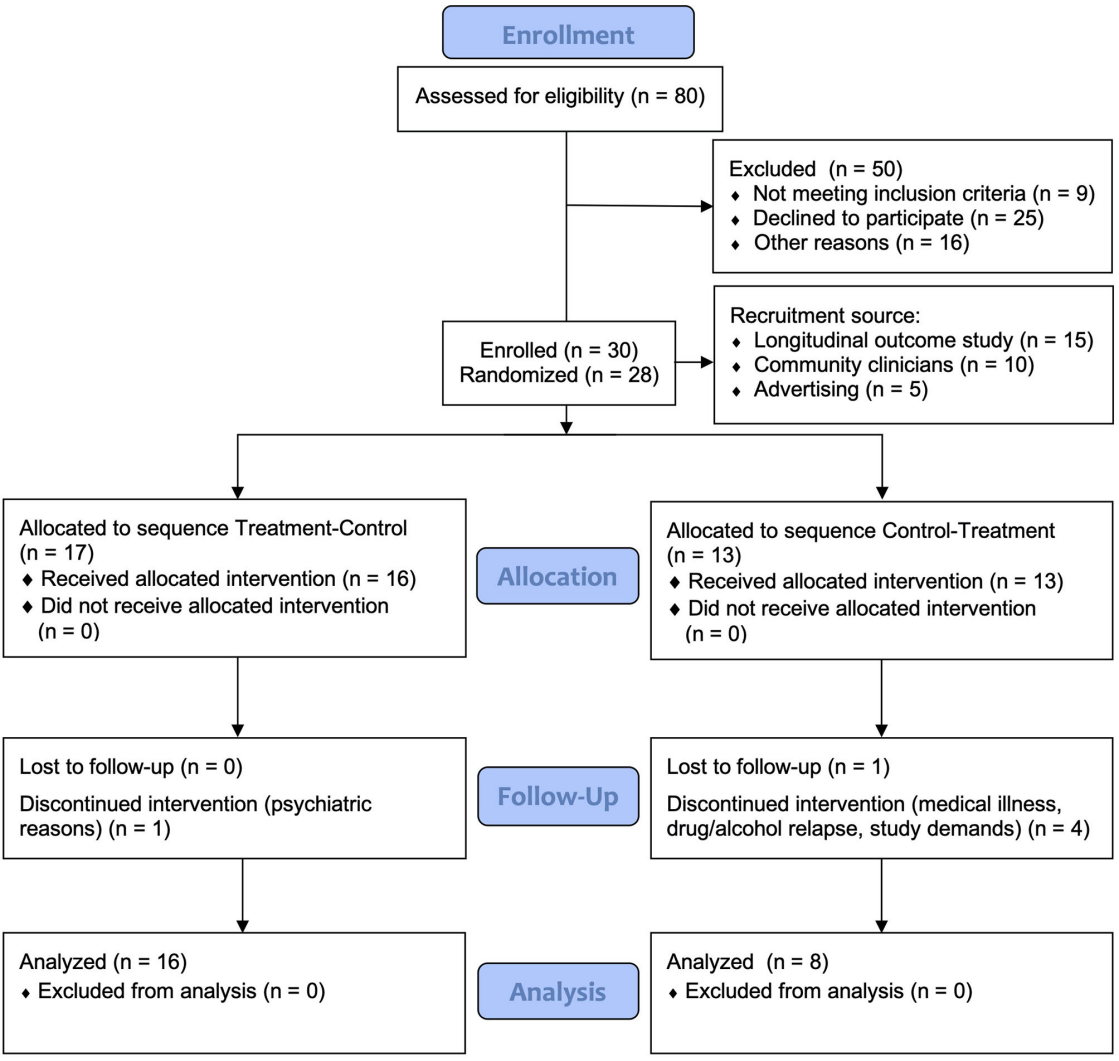
CONSORT flow diagram of recruitment and participant retention. The first two participants were not randomized as they were part of an initial two-case pilot trial.

Participants were 30 individuals enrolled with mild-severe TBI or stroke sustained at least 3 months earlier, living in the community. Inclusion criteria included history of documented mild to severe TBI, or stroke, and self-reported significant fatigue (Fatigue Severity Scale ≥4) (34). Individuals with comorbid psychiatric disorder requiring hospitalization were excluded. Other exclusion criteria included presence of another medical illness accounting for fatigue, including other neurological disorders, pre-injury sleep disorders or chronic fatigue syndrome, presence of visual impairments potentially affecting sensitivity and response to light, recent transmeridian travel, current use of prescribed and over-the-counter sleep medications and inability to give informed consent as assessed by the referring clinician or recruiting neuropsychologist. The use of antidepressants was permitted provided a stable dosage was maintained from baseline across the course of the study. Study recruitment and retention is found in the CONSORT chart in Figure 1.

### Outcome Measures

#### Primary Outcome

The findings of the RCT (17) showed no statistically significant effect on fatigue, measured using the *Brief Fatigue Inventory (BFI)* (35), but changes were in the expected direction, with medium effect size. The relatively small sample size in this pilot trial likely impacted on statistical power to detect a treatment effect. Nevertheless, we included our primary fatigue outcome measure in the current study to examine potential factors that may be worthy of investigation in future studies.

#### Secondary Outcomes

In light of our findings that demonstrated light therapy was effective in significantly improving self-reported sleep quality, psychomotor vigilance and productive activity, the following measures were selected as additional study outcomes, obtained at the end of Treatment and Control assessments. *Pittsburgh Sleep Quality Index (PSQI)*: assessed subjective global sleep quality (36). *Psychomotor Vigilance Task (PVT)*: was used to measure sustained attention and reaction time once during the day (between 10 a.m. and 5 p.m.), with mean RT as the measure (37). *Activity Diary:* A customized activity questionnaire was used to calculate minutes spent on productive (physical or mental) activity, rest and sleep in four time blocks across the course of the day (9 a.m. to 9 p.m.), with percentage of daily productive activity calculated.

#### Predictor Variables

Measures were obtained at baseline prior to exposure to Condition 1 lighting. Participant demographic, medication and injury-related information were obtained from medical files and participant interview at baseline. Variables included age, sex, time post-injury, injury type (TBI or stroke), work status (unemployed, part time or full time), and use of antidepressant medication. Duration of post-traumatic amnesia (PTA) was used as a measure of injury severity.

Baseline fatigue *(BFI)*, daytime sleepiness *(Epworth Sleepiness Scale–ESS)* (38), depression symptoms (Hospital Anxiety and Depression Scale - HADS-D) (39), chronotype *(Horne-Ostberg Morningness-Eveningness Questionnaire - MEQ)* (40) and eye color, as captured by the *Fitzpatrick Skin Type Assessment* (41), were used as predictor variables.

### Data Analysis

Data were analyzed using RStudio Version 1.3.959 (42) and *lme4* (43). All variables met assumptions of linearity, homogeneity of variance and had normally distributed residuals. A linear mixed-model analysis was used to model each outcome variable as a linear function of treatment (i.e., treatment or control), period (i.e., differences between condition 1 and condition 2 for treatment-control and control-treatment) and sequence (i.e., participants allocated treatment-control vs. participants allocated control-treatment), with participant included as a random variable. The analysis controlled for baseline scores of the respective outcome. The key analysis comprised modeling each predictor as an interaction with treatment, permitting examination of whether the effect of treatment changed as a function of predictor values. Predictors were considered significant if this interaction revealed a *p-*value <0.05. Given the small sample in this pilot trial, effect sizes were also reported.

## Results

### Participants

Thirty participants enrolled in the study; 24 participants completed the study protocol. During the study, six participants withdrew, two during the baseline period, three during Condition 1 (Control) and one during Condition 1 (Treatment). Table 1 presents the demographic and clinical data for participants at baseline, displayed by treatment sequence. There were no significant differences between the groups in baseline demographics, injury-related or clinical variables. All individuals reported clinically significant fatigue (FSS ≥ 4) at inclusion. The sample comprised predominantly participants with severe injury (PTA > 7 days; 53%). The most common mechanism of injury was TBI by motor vehicle accident (21%), and stroke (21%).

**Table 1:** Demographic and clinical characteristics by treatment sequence at baseline.

**Baseline variables**	**Treatment-control (*****n*** **=** **16)**^a^		**Control-treatment sequence (*****n*** **=** **8)**^a^		**Total (*****N*** **=** **24)**^a^	
	***M***	***SD***	***M***	***SD***	***M***	***SD***
Age (years)	43.13	10.67	46.75	13.13	44.33	11.39
Sex (female)	7	(43.75%)	3	(37.50%)	10	(41.66%)
Injury type (TBI)	14	(87.50%)	5	(62.50%)	19	(79.17%)
Time post-injury (years)	9.53	8.89	11.67	10.68	10.24	9.34
Range	1.16–26.00		3.08–31.00		1.16–31.00	
PTA^b^	28.35	25.96	15.80	24.87	24.86	25.59
Antidepressant medication (Yes)	3	(18.75%)	2	(25.00%)	5	(20.83%)
**Work status**						
PT	6	(37.50%)	4	(50.00%)	10	(41.67%)
FT	6	(37.50%)	1	(12.50%)	7	(29.17%)
None	4	(25.00%)	3	(37.50%	7	(29.17%)
Fitzpatrick skin type assessment (eye color)^c^	1.87	1.13	1.75	1.04	1.83	1.07
BFI	5.33	1.75	6.75	0.85	5.81	1.64
FSS	5.07	1.27	5.85	0.64	5.33	1.15
ESS	7.69	3.89	10.25	5.20	8.54	4.43
PSQI	7.81	4.09	9.38	4.03	8.33	4.05
HADS (depression)	6.31	3.40	8.25	3.01	6.96	3.34
MEQ	60.19	11.68	51.50	7.65	57.29	11.15
PVT: mean RT^b^	345.82	89.11	332.68	62.96	321.58	52.41
Productive activity (%)	85.82	12.28	84.17	9.67	85.16	10.96

*BFI, Brief Fatigue Inventory; ESS, Epworth Sleepiness Scale; FSS, Fatigue Severity Scale; FT, Full time; HADS, Hospital Anxiety and Depression Scale; MEQ; Morningness-Eveningness Questionnaire; PT, Part time; PTA, Post-traumatic Amnesia; PVT, Psychomotor Vigilance Task*.

*There were no significant differences in outcomes between sequences at Baseline*.

a *Data are mean (M) and standard deviation (SD), or percentage values, of participant demographics and baseline characteristics shown for each treatment group*.

b *N = 18; results not available for some participants due to equipment failure or missing data. No PTA for stroke participants*.

c *N = 23*.

### Factors Associated With Reduced Fatigue

**Figure 2:**
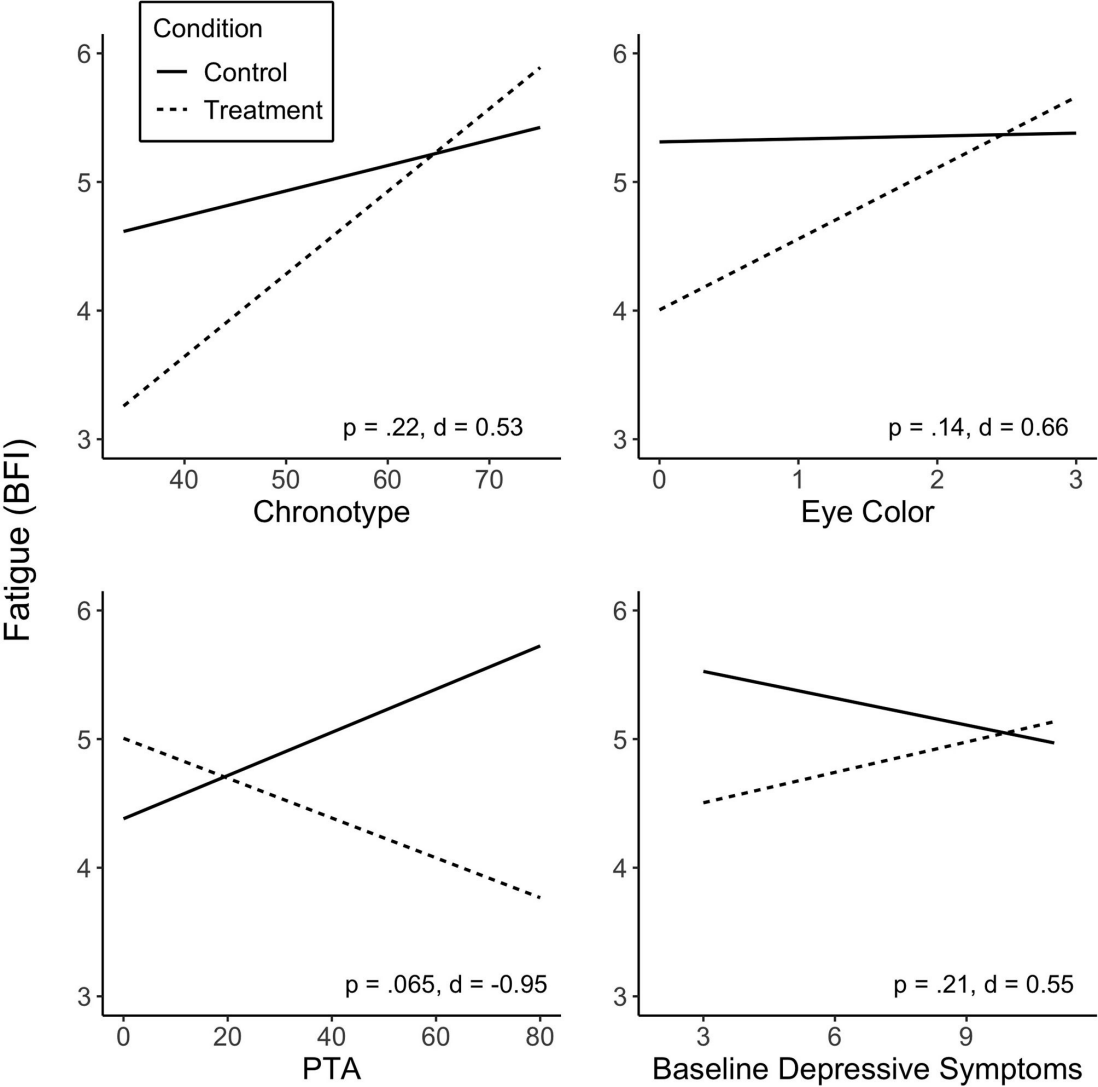
Predicted values of fatigue (BFI) from chronotype **(A)**, eye color **(B)**, injury severity (PTA duration) **(C)** and baseline depressive symptoms **(D)**. Predictors did not significantly moderate treatment effect on fatigue, but showed medium and large effect sizes. Treatment sequences are combined in figures. Significance (*p*) and effect size (Cohen's *d*) represented are for predictor-treatment interaction. BFI score range 1–10; score of ≥4 suggests clinically significant fatigue.

No predictor was found to significantly moderate the effect of treatment on fatigue (*p* > 0.05; see Supplementary Table 1 for summary of the predictors in linear mixed-model analyses). Some variables did, however, show medium to large effect sizes. Specifically, the interaction between chronotype and Treatment revealed a medium effect size (*d* = 0.53), in that individuals who were more evening type experienced a greater reduction in fatigue on the BFI during Treatment. Lighter eye color (blue, gray or green), whilst non-significant, was associated with greater Treatment response in fatigue, showing a medium effect size (*d* = 0.66). PTA showed a large effect size (*d* = 0.95); greater injury severity at baseline was associated with greater fatigue reduction during the Treatment condition. The association of baseline depressive symptoms with fatigue levels was not statistically significant, but showed a medium effect size (*d* = 0.55), associating lower baseline symptoms with greater reduction in fatigue. There was no significant association observed between sequence (Treatment-Control or Control-Treatment) or baseline fatigue levels and fatigue reduction in response to treatment. Figure 2 shows predicted values of BFI according to chronotype, eye color, PTA and baseline depressive symptoms.

### Predictors of Improved Sleep Quality

**Figure 3:**
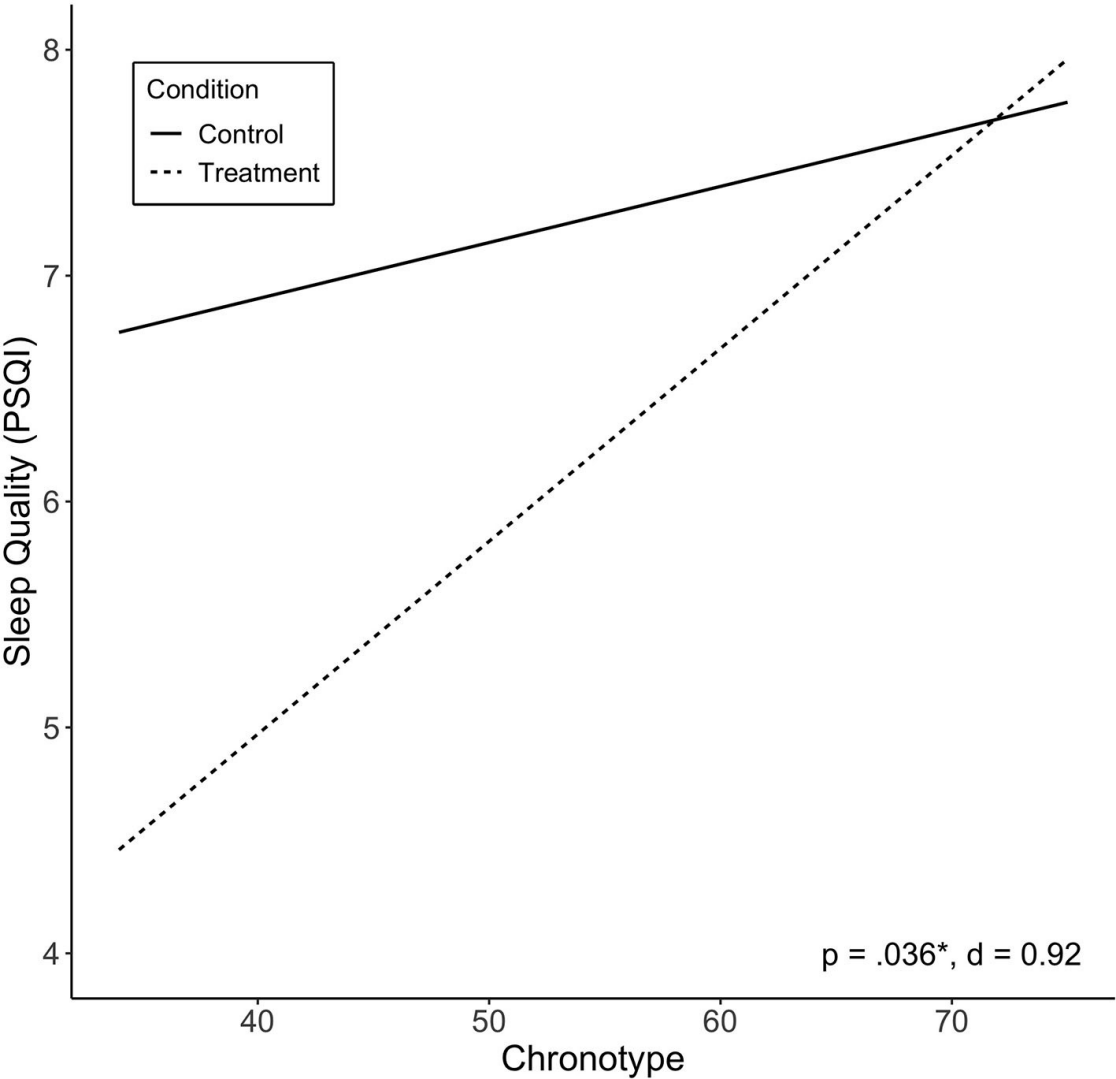
Predicted values of sleep quality (PSQI) by chronotype. Chronotype significantly moderated treatment effect on sleep quality, and showed large effect size. Treatment sequences are combined in figure. Significance (*p*) and effect size (Cohen's *d*) represented are for predictor-treatment interaction.

Of the independent variables examined, we found chronotype to be significantly associated with treatment response in sleep quality (PSQI; *p* = 0.036). Lower scores (more evening type) were associated with greater improvement in sleep quality (Figure 3). Other variables were not found to be significantly associated with change in sleep quality, including baseline PSQI scores. There was no significant effect of treatment sequence.

### Predictors of Reaction Time Response

**Figure 4:**
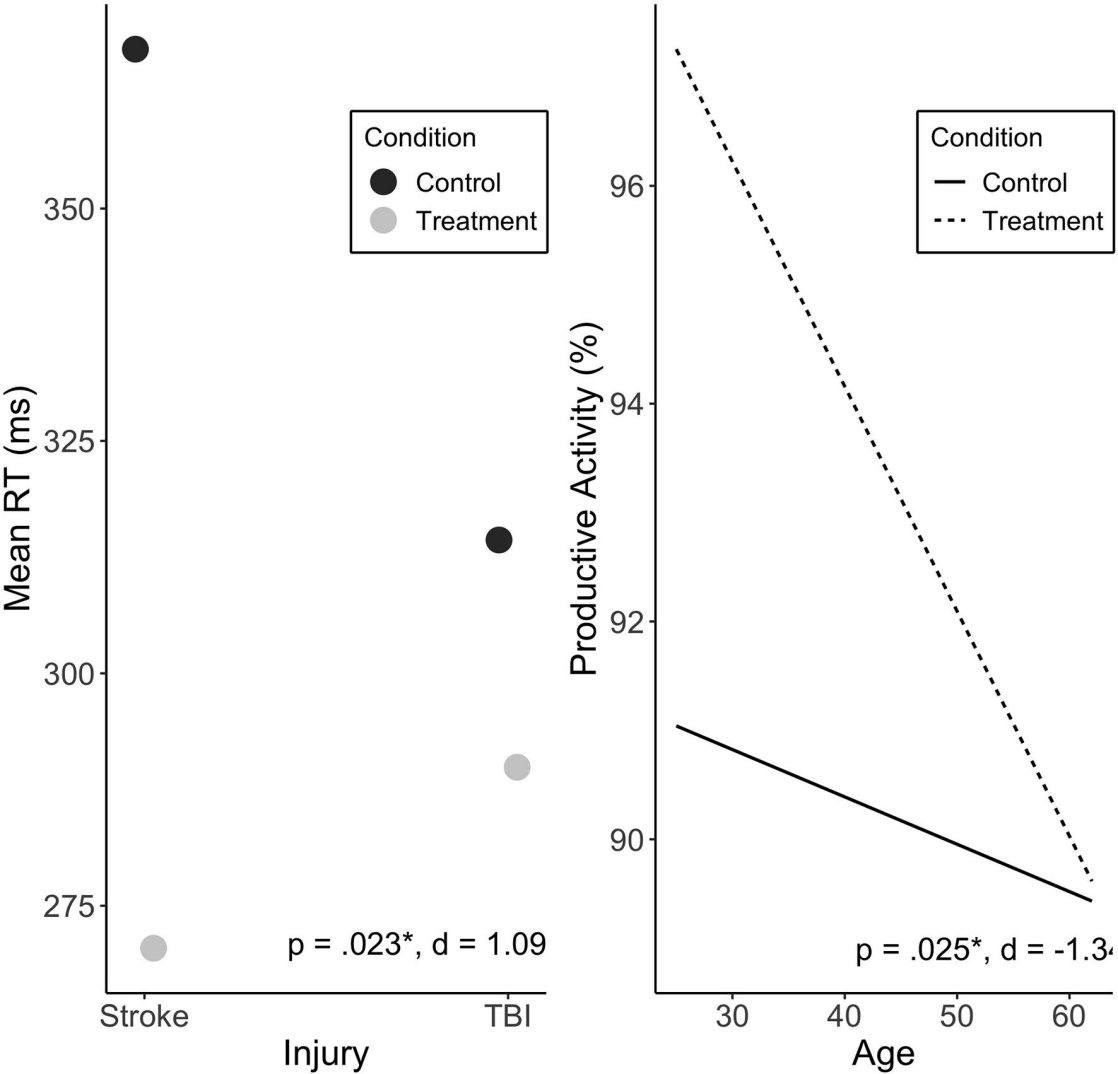
Predicted values of mean RT (ms) by injury type **(A)**, and productive activity (%) by age **(B)**. Injury type significantly moderated treatment effect on mean RT, and showed large effect size. Age significantly moderated treatment effect on productive activity, with large effect. Treatment sequences are combined in figure. Significance (*p*) and effect size (Cohen's *d*) represented are for predictor-treatment interaction.

Injury type moderated the effect of Treatment on mean RT (*p* = 0.023). Stroke participants were found to exhibit a greater Treatment response than participants with TBI (Figure 4). There was no significant effect of treatment sequence. Other variables were not associated with change in mean RT, including baseline RT scores (see Supplementary Table 1).

### Predictors of Increase in Productivity Levels

Age was found to significantly predict change in productive activity levels during Treatment (*p* = 0.025), with no significant effect of sequence. Younger individuals exhibited a more positive Treatment response reflected in increased activity levels (see Figure 4).

## Discussion

This study aimed to identify factors associated with response to a novel in-home light therapy for fatigue and sleep disturbance in individuals with TBI or stroke. We examined the association of a range of variables with changes in the primary outcome of fatigue, and secondary outcomes of sleep disturbance, psychomotor vigilance and productive activity in response to this treatment, including demographic and injury-related variables, baseline symptoms, and factors that may have impacted an individual's sensitivity to light including chronotype, eye color and use of antidepressant medication.

The clinical trial (17) did not show a statistically significant benefit on the primary outcome, fatigue. There were also no variables that were significantly associated with changes in fatigue, although several factors did show associations with medium or large effect sizes. Evening chronotype, lighter eye color, greater injury severity as indicated by longer PTA, and lower baseline depressive symptoms were associated with greater post-treatment reduction in fatigue. These preliminary findings are important in informing future light intervention studies.

Evening chronotype was associated with greater Treatment response in sleep quality, and a trend toward reduced fatigue. There are a number of possible reasons for this relationship. Chronotype is a behavioral phenotype resulting from an approximately equal contribution of the endogenous circadian clock and the sleep homeostat (the two-process model of sleep-wake regulation). A tendency for eveningness, for example, could result from a later circadian clock phase, a slower homeostatic build-up of sleep pressure, or both (44). The benefits of light treatment could also be mediated through both of these processes. The greater light response observed for evening types could be a result of a phase advance (earlier) shift in the circadian clock due to less exposure to blue-enriched light that would phase delay in the evening, and exposure to blue-enriched light to phase advance in the morning (45), which would in turn better realign the natural alertness rhythm with day, and sleep with night, improving daytime function. A reduction in the direct alerting effects of light in the 3 h prior to sleep through exposure to blue-depleted light would also have a direct effect on the ability to fall asleep, and sleep quality, as shown previously (46–48). This chronotype-treatment interaction has also been observed in other groups, in treatment-resistant inpatients with depression using bright light therapy (29), and in bipolar patients using combined total sleep deprivation/morning light therapy (30), who both showed those with evening chronotype exhibited greater treatment response. The current study cannot quantify the relative contributions of these mechanisms but future studies could include outpatient circadian phase or waking EEG measures to try and do so.

The association between lighter iris color and better light therapy Treatment response in fatigue is consistent with previous work that demonstrated increased sensitivity of another “non-visual” response to light, melatonin suppression response, in individuals with light-colored, compared to dark-colored, eyes (27). This is thought to be due to intraocular light scattering, called straylight, which is dependent on pigmentation of the eye, and found to be greater in blue-eye individuals than dark-eyed individuals (49). Whilst this personal factor may not be warranted as a selection criterion for suitability to undertake light therapy, it may help explain variability across individuals.

The finding that greater injury severity (measured by PTA, in TBI participants only) was associated with a larger treatment response was not in line with our hypothesis. If confirmed, this finding is promising as it may mean that individuals with more severe injuries are able to benefit from this relatively non-invasive intervention. Furthermore, injury type (i.e., stroke or TBI) was found to significantly predict change in mean RT. Specifically, stroke participants exhibited a greater Treatment response than those with TBI. While of interest, the finding should be considered preliminary as, to our knowledge, this is only known light therapy study to examine patients with two separate injury types and given the heterogeneity in injury and the small sample of stroke participants in the current study, the result should be treated with caution until replicated. Unfortunately, our groups were unbalanced (*n* = 16 in Treatment-Control and *n* = 8 in Control-Treatment), which was due to the first two participants not being randomized, as part of a pilot trial, and withdrawals in the latter sequence. Increasing participant numbers in a larger trial will assist in balancing these groups.

The trend toward reduced Treatment effect on fatigue in those with higher baseline depressive symptoms is supported by literature demonstrating decreased melatonin suppression to light in those with current depression (50). The association between a current depressive episode and reduced sensitivity to light may mean that those with depressive symptoms might experience reduced benefit from the alertness-enhancing effects of light treatment. The causal mechanism for this hyposensitivity to light is unknown.

Age significantly predicted change in productive activity levels during Treatment, with younger individuals exhibiting a stronger Treatment effect. It is plausible that younger individuals in our sample had greater capacity for change in physical activity, as they were possibly more mobile than older participants. Lam also observed younger age to be significantly associated with a better light treatment response on a measure of depression, in a sample of individuals with SAD, with age accounting for 10% of the variance in response (21). This relationship could also be due to an increase in lens yellowing with age, which may reduce the transmission of the alerting short-wavelength light during the day (51), although the magnitude of this effect remains under debate (52).

Although the current study examined a range of predictive variables, there are likely to be other factors which may have influenced response to the in-home light therapy in this study. In a systematic review and meta-analysis on the effects of light therapy on sleep problems, van Maanen et al. highlighted that treatment characteristics may impact treatment response including “number of treatment days, daily treatment duration and intensity and spectral characteristics of light” [(31), p. 53]. They found treatment for insomnia to be significantly moderated by light intensity, in that larger effects were found for studies using higher light intensity. A study examining factors predicting therapeutic outcomes with DSWPD also echoed the sentiment that consistency in exposure is a key factor in treatment efficacy. They found the number of light therapy days using a light box predicted both earlier sleep onset and earlier sleep offset, and emphasized the importance of daily usage (18). The 8-week treatment duration was a strength in our study design, as many outcomes appeared to show improvement between Weeks 4 and 8. The use of the entire ambient day-evening light environment as the therapeutic vehicle, rather than limited exposure to a light box, was also a strength, although the trade-off between these approaches remains to be tested directly; is it better to have shorter-duration but more controlled and higher intensity light box exposure in the morning over fewer weeks, compared to “all-day” ambient but less well-controlled exposure over a longer (life)time? Future studies should examine the change in spectral characteristics (e.g., melEDI) as a predictive factor of treatment response, and even longer treatment durations given the passive nature of the intervention and low patient burden.

Recent literature suggests there is significant variability in light-induced melatonin suppression across individuals, with some individuals reaching 50% suppression with exposure to as little as 6 lux light stimuli, whilst others requiring up to 350 lux to achieve the same degree of suppression (53). With these findings in mind, it is plausible that the current study's therapeutic conditions during daytime were not sufficiently intense to adequately suppress melatonin for some participants, or that individuals with greater sensitivity may have suffered from over-exposure (and too much melatonin suppression) to light in the evening. Measuring melatonin suppression may be a desirable addition for future studies examining the efficacy of light therapy.

The current study examined factors associated with treatment response in a pilot study of a novel home-based light therapy. The results showed that chronotype was able to significantly predict Treatment effect on sleep quality, injury type could predict mean RT, and age could predict productive activity. Specifically, evening chronotype, stroke injury, and younger age were associated with greater treatment response. Whilst there were no variables found to be significantly associated with change in our primary outcome of fatigue, some variables revealed promising effect sizes, including chronotype, eye color, injury severity as measured by PTA, and baseline depressive symptoms. A larger study including more stroke patients and other predictive factors is warranted. The results of this pilot study are encouraging in establishing a potential novel therapeutic approach. Understanding potential factors that may alter clinical outcomes is vital to understand the generalizability of the treatment, and will enable clinicians to apply a more targeted approach to prescribing light therapy and assessing suitability on a range of patient factors that speak to the likelihood of a positive treatment response.

## Data Availability Statement

The raw data supporting the conclusions of this article will be made available by the authors, without undue reservation.

## Ethics Statement

The studies involving human participants were reviewed and approved by Epworth HealthCare Human Research Ethics Committee and Monash University Human Research Ethics Committee. The patients/participants provided their written informed consent to participate in this study.

## Author Contributions

JP and GS contributed to the conception and design of the study. LC performed the statistical analysis and wrote the first draft of the manuscript. SL wrote sections of the manuscript. All authors contributed to manuscript revision, read, and approved the submitted version.

## Conflict of Interest

SR is the Program Leader for the CRC for Alertness, Safety and Productivity, Australia; Director (now Chair) of the Sleep Health Foundation. He has received grants from Vanda Pharmaceuticals, Philips Respironics, Cephalon, Rio Tinto, BHP Billiton and Shell which are not related to this paper. SR has received equipment support and consultancy fees through his institution from Optalert, Compumedics, Teva Pharmaceuticals, and Circadian Therapeutics, which are not related to this paper. SL has had a number of commercial interests in the last 3 years (2018–20). His interests were reviewed and managed by Brigham and Women's Hospital and Partners HealthCare in accordance with their conflict of interest policies. No interests are directly related to the research or topic reported in this paper but, in the interests of full disclosure, are outlined here. SL has received consulting fees from the BHP Billiton, EyeJust Inc., Noble Insights, Rec Room, Six Senses, Stantec and Team C Racing; and has current consulting contracts with Akili Interactive; Apex 2100 Ltd.; Consumer Sleep Solutions; Headwaters Inc.; Hintsa Performance AG; KBR Wyle Service, Light Cognitive; Lighting Science Group Corporation/HealthE; Look Optic; Mental Workout/Timeshifter and View Inc. He has received honoraria and travel or accommodation expenses from Emory University, Estée Lauder, Ineos, MIT, Roxbury Latin School, and University of Toronto, and travel or accommodation expenses (no honoraria) from IES, Mental Workout, Solemma, and Wiley; and royalties from Oxford University Press. He holds equity in iSleep pty. He has received an unrestricted equipment gift from F. Lux Software LLC, a fellowship gift from Stockgrand Ltd. and holds an investigator-initiated grant from F. Lux Software LLC and a Clinical Research Support Agreement with Vanda Pharmaceuticals Inc. He is an unpaid Board Member of the Midwest Lighting Institute (non-profit). He was a Program Leader for the CRC for Alertness, Safety and Productivity, Australia, through an adjunct professor position at Monash University (2015-2019). He has served as a paid expert in legal proceedings related to light, sleep and health. The remaining authors declare that the research was conducted in the absence of any commercial or financial relationships that could be construed as a potential conflict of interest.
