# Supplementary material for: Factors Associated With Response to Pilot Home-Based Light Therapy for Fatigue Following Traumatic Brain Injury and Stroke
Source: Front Neurol. 2021 Jul 15;12:651392. doi: 10.3389/fneur.2021.651392 (PMC8319544; doi:10.3389/fneur.2021.651392)
Supplement: Supplementary file 1 [file Table_1.DOCX]

Supplementary Material

**Supplementary Table 1**

**Mixed-model results by outcome and predictor**

| Outcome | Predictor | Treatment effect estimate (95% CI) | *t* statistic | Effect size (*d)* | *p* value |
| --- | --- | --- | --- | --- | --- |
| BFI | Sex [Male] | -0.33(-1.84-1.19) | -0.42 | -0.19 | .67 |
|  | Age | 0.03 (-0.04-0.10) | 0.90 | 0.39 | .37 |
|  | Injury [TBI] | 0.19 (-1.74-2.12) | 0.19 | 0.08 | .85 |
|  | Chronotype | 0.04 (-0.03-0.12) | 1.22 | 0.53 | .22 |
|  | Eye Color^a^ | 0.53 (-0.18-1.23) | 1.47 | 0.66 | .14 |
|  | Work Status [None] | -0.28 (-2.34-1.78) | -0.27 | -0.12 | .79 |
|  | Antidepressant [Yes]^a^ | 0.53 (-2.47-1.27) | -0.63 | -0.28 | .53 |
|  | PTA^b^ | -0.03 (-0.07-0.00) | -1.85 | -0.95 | .065 |
|  | Time Post Injury | 0.02 (-0.06-0.10) | 0.47 | 0.21 | .64 |
|  | Baseline BFI | -0.07 (-0.58-0.45) | -0.25 | -0.11 | .80 |
|  | Baseline ESS | 0.10 (-0.08-0.27) | 1.11 | 0.48 | .27 |
|  | Baseline Depressive Symptoms | 0.15 (-0.08-0.38) | 1.26 | 0.55 | .21 |
| PSQI | Sex [Male] | 0.16 (-1.11-1.44) | 0.25 | 0.11 | .80 |
|  | Age | 0.02 (-0.04-0.07) | 0.53 | 0.23 | .59 |
|  | Injury [TBI] | -0.48 (-2.09-1.12) | -0.59 | -0.26 | .56 |
|  | Chronotype | 0.06 (0.00-0.12) | 2.10 | 0.92 | .036* |
|  | Eye Color^a^ | 0.00 (-0.62-0.62) | 0.00 | < .001 | > .99 |
|  | Work Status [None] | -0.35 (-2.06-1.37) | -0.40 | -0.18 | .69 |
|  | Antidepressant [Yes]^a^ | -0.99 (-2.52-0.54) | -1.26 | -0.56 | .21 |
|  | PTA^b^ | -0.01 (-0.04-0.02) | -0.72 | -0.37 | .47 |
|  | Time Post Injury | 0.01 (-0.06-0.07) | 0.16 | 0.07 | .87 |
|  | Baseline BFI | -0.01 (-0.44-0.42) | -0.05 | -0.02 | .96 |
|  | Baseline ESS | 0.02 (-0.13-0.17) | 0.26 | 0.12 | .79 |
|  | Baseline Depressive Symptoms | 0.07 (-0.13-0.27) | 0.67 | 0.29 | .50 |
|  | Baseline PSQI | 0.01 (-0.15-0.17) | 0.08 | 0.04 | .94 |
| Mean RT^b^ | Sex [Male] | 1.58 (-36.38-39.54) | 0.08 | 0.07 | .94 |
|  | Age | 0.36 (-1.22-1.94) | 0.44 | 0.32 | .66 |
|  | Injury [TBI] | 72.26 (9.90-134.63) | 2.27 | 1.09 | .023* |
|  | Chronotype | 0.55 (-1.51-2.62) | 0.53 | 0.35 | .60 |
|  | Eye Color^c^ | -10.69 (-27.64-6.25) | -1.24 | -0.85 | .22 |
|  | Work Status [None] | -13.37 (-60.16-33.42) | -0.56 | -0.41 | .58 |
|  | Antidepressant^c^ [Yes] | -7.08 (-46.97-32.82) | -0.35 | -0.22 | .73 |
|  | PTA^d^ | 0.16 (-0.70-1.01) | 0.37 | 0.17 | .71 |
|  | Time Post Injury | 0.82 (-1.04-2.68) | 0.86 | 0.63 | .39 |
|  | Baseline BFI | 2.24 (-10.68-15.17) | 0.34 | 0.24 | .73 |
|  | Baseline ESS | 2.50 (-2.61-7.61) | 0.96 | 0.59 | .34 |
|  | Baseline Depressive Symptoms | 2.10 (-3.84-8.04) | 0.69 | 0.50 | .49 |
|  | Baseline Mean RT | 0.01 (-0.38-0.41) | 0.07 | 0.05 | .95 |
| Productive Activity^e^ | Sex [Male] | 0.91 (-3.31-5.12) | 0.42 | 0.25 | .67 |
|  | Age | -0.16 (-0.31- -0.02) | -2.23 | -1.34 | .025* |
|  | Injury [TBI] | -1.79 (-7.57-3.99) | -0.61 | -0.36 | .54 |
|  | Chronotype | -0.10 (-0.33-0.14) | -0.81 | -0.48 | .42 |
|  | Eye Color^d^ | -0.23 (-2.10-1.64) | -0.24 | -0.15 | .81 |
|  | Work Status [None] | 1.07 (-4.28-6.42) | 0.39 | 0.25 | .70 |
|  | Antidepressant [Yes] | 1.22 (-4.59-7.02) | 0.41 | 0.24 | .68 |
|  | PTA^f^ | -0.05 (-0.16-0.06) | -0.88 | -0.62 | .38 |
|  | Time Post Injury | 0.03 (-0.19-0.24) | 0.23 | 0.14 | .82 |
|  | Baseline BFI | 0.17 (-1.46-1.80) | 0.21 | 0.12 | .84 |
|  | Baseline ESS | -0.29 (-0.72-0.13) | -1.34 | -0.80 | .18 |
|  | Baseline Depressive Symptoms | 0.24 (-0.34-0.82) | 0.82 | 0.49 | .41 |
|  | Baseline Productive Activity | 0.02 (-0.17-0.20) | 0.17 | 0.10 | .86 |

*Notes.* Predictors were entered into models as interactions with Treatment. **p* < .05.

^a^N = 23. ^b^N = 18. ^c^N = 17. ^d^N = 14. ^e^N = 15. ^f^N = 11.
